# Comprehensive analysis of the prognostic value and immunological role of *IDO1* gene in pan-cancer

**DOI:** 10.1186/s40001-024-01766-y

**Published:** 2024-03-27

**Authors:** Kaili Lin, Yongfeng Wang, Fangyu Liu, Taifu You, Xiongxiong Liu, Runzhang Liu, Zeyang Li, Chunyu Zhen, Yunxia Zhang, Xingguang Liu, Hui Cai

**Affiliations:** 1 Lanzhou First People’s Hospital, Lanzhou, 730000 Gansu China; 2 Present Address: The First Clinical Medical College of Lanzhou University, Lanzhou, 730000 Gansu China; 3 General Surgery Clinical Medical Center, Gansu Provincial Hospital, Lanzhou, 204 Donggang West Road, Lanzhou, 730000 Gansu China; 4 Key Laboratory of Molecular Diagnostics and Precision Medicine for Surgical Oncology in Gansu Province, Gansu Provincial Hospital, Lanzhou, 730000 Gansu China; 5 NHC Key Laboratory of Diagnosis and Therapy of Gastrointestinal Tumor, Gansu Provincial Hospital, Lanzhou, 730000 Gansu China

**Keywords:** *IDO1*, Prognosis, Bioinformatics analysis, Cancers

## Abstract

**Objective:**

It has been demonstrated that IDO1, a target of immune checkpoint inhibition, functions as an oncogene in the majority of human malignancies. IDO1’s function in human pan-cancers hasn’t been thoroughly studied, though.

**Materials and methods:**

The Kaplan–Meier (K-M) and COX analyses were applied to the survival analysis. Furthermore, we used Spearman’s correlation analysis to examine the associations between *IDO1* and microsatellite instability (MSI), DNA methyltransferases (DNMTs), tumor mutational burden (TMB), the associated genes of mismatch repair (MMR), and immune checkpoint biomarkers. Moreover, immunohistochemical analysis and qRT-PCR were used to evaluate IDO1’s expression in pan-cancer cells.

**Results:**

The findings of this study reveal that *IDO1* has abnormal expression in a number of malignancies and is related to the prognosis for UVM, LGG, KIRP, GBM, LAML, OV, READ, MESO, SARC, SKCM, and HNSC. Furthermore, the aberrant *IDO1* expression was connected to the TMB, MSI, MMR, drug sensitivity, immune cells infiltrating, and tumor immune microenvironment across a variety of cancer types. The PCR results showed that in contrast to normal cells, IDO1 was found to be significantly highly expressed in breast cancer cells and hepatocellular carcinoma cells, and significantly lowly expressed in gastric cancer cells.

**Conclusion:**

The clinical treatment of IDO1 is now better supported by a theoretical basis and guidelines provided by our study.

**Supplementary Information:**

The online version contains supplementary material available at 10.1186/s40001-024-01766-y.

## Introduction

A growing number of people are being diagnosed with cancer and dying from it. According to the World Health Organization (WHO) study from 2022, cancer accounts for approximately 16% of fatalities worldwide and is one of the main causes of mortality, particularly for those under the age of 70 [1]. Despite advances in cancer detection and therapy, low survival rates are still caused by several issues, including cancer recurrence and medication resistance [2]. Finding new techniques for cancer diagnosis and treatment is crucial [3]. A combination immunotherapy strategy is more likely to improve survival than a single-drug therapeutic approach, according to preclinical and clinical studies [4, 5]. Therefore, it is crucial to look for new cancer markers to more accurately and early identify the clinical stage, metastasis, and prognosis of most malignancy [6, 7].

Numerous studies have demonstrated that targeting the immunometabolic enzyme indoleamine 2,3 dioxygenase 1 (*IDO1*) is highly expressed in multiple types of human cancer. *IDO1* catalyzes the degradation of tryptophan (Trp) to kynurenine (Kyn). The *IDO1* gene can be induced by interferon and is associated with mediating potent immunosuppressive effects in cancer [8, 9]. *IDO1* is overexpressed in various forms of cancer and is linked to a bad prognosis. A tumor-friendly immunological microenvironment can be created by *IDO1* and Kyn metabolites, and aryl hydrocarbon receptors can be activated as part of this process [10]. IDO1 stimulates CD4 + T regulatory cells (Treg) and myeloid-derived suppressor cells (MDSC) while suppressing local CD8 + T effector cells and natural killer cells [11].

Patients who get immune checkpoint blockade therapy may see some truly astonishing lengthy anti-cancer effects [12]. Recently discovered immunological checkpoint inhibitors have been approved for use in clinical settings. One of the most effective methods for finding new cancer drugs is the development of immune checkpoint inhibitors as anticancer therapeutic alternatives in recent years [13]. *IDO1* inhibitors have been the focus of significant research over the past 10 years. *IDO1* inhibitors have been identified and are currently investigated in clinical studies, including Indoximod, Epacadostat, BMS-986205, and PF-06840003 [14–19]. *IDO1* has been linked to mediating strong immunosuppressive effects in cancer and is interferon-inducible [20]. Combining an *IDO1* inhibitor with a checkpoint inhibitor is a promising way to increase the number of patients who can receive immunotherapies because it has been demonstrated that *IDO1* takes part in the mechanisms of resistance to checkpoint inhibitors [21]. Strong evidence exists that a combination approach can offer synergistic effects, despite mounting evidence that a treatment targeting *IDO1* alone is ineffective [8, 22–25]. *IDO1* is being used as an adjuvant medication in a growing number of ongoing clinical trials in conjunction with other cancer therapy methods due to the poor efficacy of single medicines [26]. *IDO1* inhibitors have so far been established, examined, and filtered in disease-related preclinical models [26, 27]. The *IDO1* pathway modulator Indoximod (d-1-MT), the *IDO1* vaccination, and Epacadostat appears to be well tolerated by cancer patients [28–31]. The number of trials examining *IDO1* inhibition in cancer therapy is still increasing since it has been established that targeting *IDO1* is secure and well-tolerated [8].

In this study, we combined The Cancer Genome Atlas (TCGA) and Genotype-Tissue Expression (GTEx) to investigate abnormal expression and to assess its prognostic value in multiple cancer types, providing a comprehensive picture of the prognostic value and alteration of the *IDO1* gene in pan-cancer. More importantly, we investigated the relationship between *IDO1* expression and various tumor immunity, microsatellite instability (MSI), tumor mutation burden (TMB), tumor immune microenvironment (TME), and tumor-infiltrating immune cells (TIICs), and further observed the correlation between *IDO1*and tumor immunotherapy.

## Methods

### Data processing and *IDO1* expression analysis

We obtained data on *IDO1* from the TCGA and the GTEX to examine the difference of *IDO1*. From the TCGA database, we collected data on somatic mutations and clinical follow-up for 33 different types of cancer. Log_2_ transformation was used to normalize all expression levels. We later used the HPA to display human protein expression in normal and tumor tissues, which allowed us to identify the expression of the IDO1 protein.

### Analysis of mismatch repair system (MMRS) and DNA methyltransferase

It is possible that DNA MMRS defects can lead to tumorigenesis [32]. As well as altering chromatin structure and gene expression, DNMTs also affect gene expression [33]. The TCGA database was used to determine the expression levels of 5 MMR genes (EPCAM, MLH1, MSH2, MSH6, and PMS2) as well as the 4 methyltransferases (DNMT3B, DNMT2, DNMT3A, and DNMT1).

### Survival and prognosis analysis

Forest plots and K-M curves were utilized to assess the relationship between IDO1 expression and patient prognosis in 33 distinct cancer types by K-M and COX regression analysis. The ROC package was then used to create ROC curves, and the area under the curve (AUC) was used to calculate the predictive power. In addition, the RMS software produced nomogram plots and calibrations for projecting OS.

### Correlations between *IDO1* expression and immune

The scores of these six TIICs in 33 tumors were retrieved from the TIMER database. You can access 10,897 TCGA samples in the TIMER database. Additionally, using Spearman correlation analyses, we evaluated the relationships between IDO1 expression and TMB, MSI, immune/stromal scores, TIICs, the related genes of immunological checkpoint marker, MMR, and DNMTs.

### Pathway study of IDO1 in pan-cancers

An analysis of gene sets obtained from Gene Set Enrichment Analysis (GSEA) website was used for this study. Gene Ontology (GO) and KEGG (Kyoto Encyclopedia of Genes and Genomes) co-expression analysis using the R-package ‘‘org.Hs.eg.db’’, ‘‘clusterProfiler’’, and ‘‘enrichplot’’ to visualize the significant positive and negative correlations of 5 pathways. Output categories were significance threshold at p and q < 0.05.

### Cell culture

Breast cell lines MCF-7, MCF-10A, and MDA-MB-231; the liver cell line L-02, HepG2, HUH-7, and SMMC-7721 liver cell lines; and the gastric cell lines GES-1, MKN-45, AGS, and MGC-803 were all incubated in RPMI-1640 supplemented with 10% FBS (Hyclone).

### RNA isolation and Q-PCR

The M5 Universal RNA Mini Kit kit's instructions were followed to extract total RNA. To confirm that the RNA concentration and purity were constant, the spectrophotometer was used to measure the absorbance values at 280 nm and 260 nm. Following the directions in the M5 Sprint qPCR RT kit with gDNA remover reverse transcription kit, RNA was reverse transcribed into cDNA. For RT-qPCR detection, we used cDNA as a template and 2 M5 HiPer SYBR Premix EsTaq (with Tli RnaseH) as a fluorescent dye. Wuhan Sevier Biotechnology Business Ltd created and created *IDO1* primers.

### Statistical analysis

An analysis of the expression of IDO1 in various tissues was conducted using Kruskal–Wallis tests and pair-t-tests. *P* < 0.05 denotes statistically significant.

## Results

### Differential expression analysis of *IDO1*

**Fig. 1:**
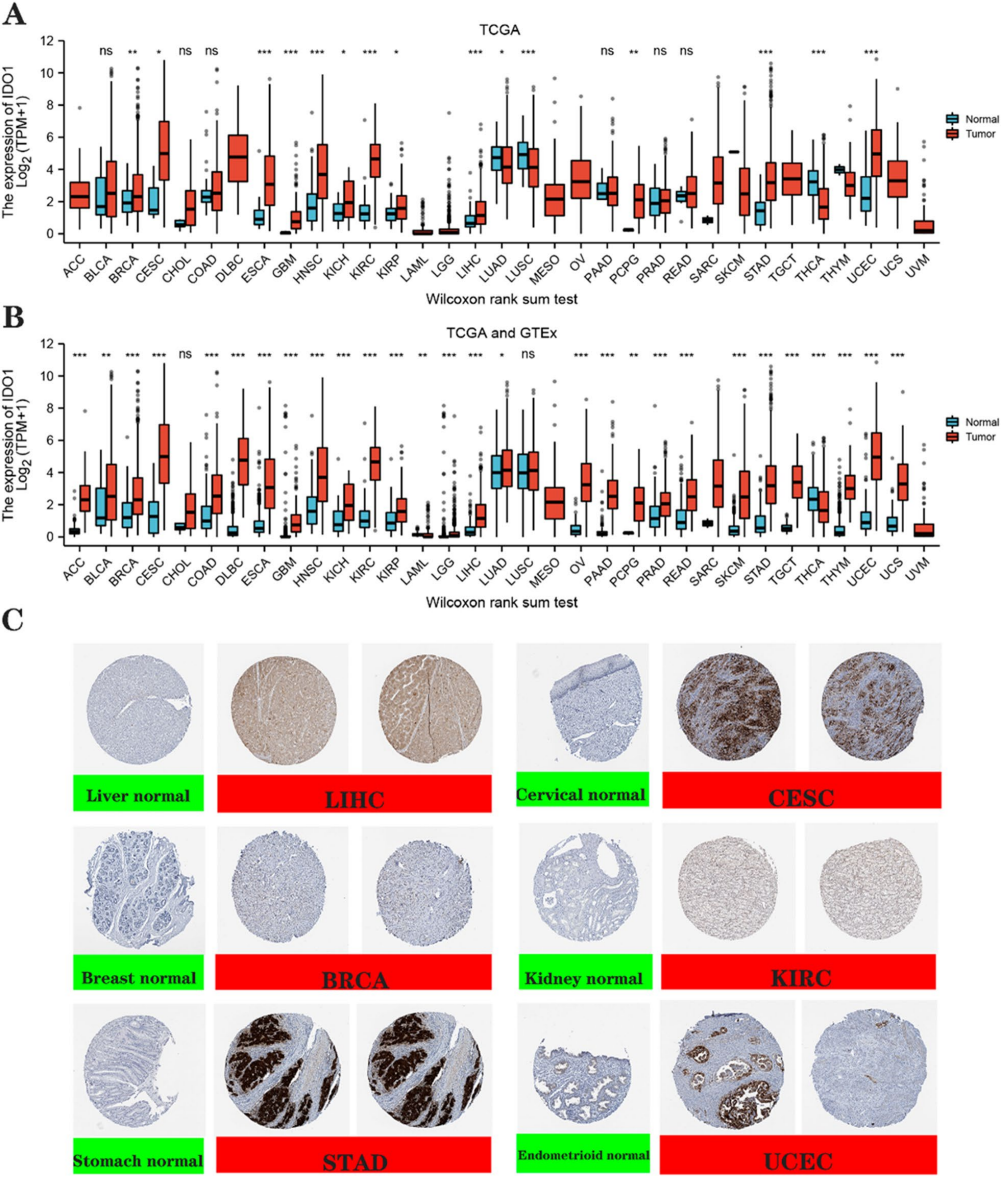
Comprehensive analysis of the differential expression of IDO1. **A** Differential IDO1 mRNA expression in TCGA database. **B** Differential IDO1 mRNA expression between TCGA and GTEX databases. *P < 0.05, **P < 0.01, ***P < 0.001. **C** Immunohistological images comparing IDO1 gene expression between normal and tumor tissues for normal (left) as well as tumor (right) in LIHC, CESC, BRCA, KIRC, STAD, and UCEC

The results showed that among the thirty-three tumors associated with gene expression, *IDO1* mRNA was highly expressed in twelve tumors in CESC, BRCA, ESCA, HNSC, GBM, KICH, KIRC, KIRP, PCPG, LIHC, STAD, and UCEC by TCGA database; especially overexpressed in CESC, KIRC, HNSC, and UCEC. IDO1 is lowly expressed in three tumors of LUAD, LUSC, and THCA (Fig. 1A and Additional file 2: Table S1).

We also examined the *IDO1* expression by integrating TCGA data with normal tissue data from the GTEx database. We discovered that 26 different tumor types over-expressed *IDO1*. In addition to the 12 types of tumors mentioned above, there are ACC, COAD, BLCA, DLBC, LGG, LUAD, PAAD, OV, PRAD, READ, TGCT, SKCM, THYM, and UCS, the combined data showed significant differences in IDO1 expression in CESC, DLBC, KIRC, OV, STAD and UCEC. Meanwhile, *IDO1* was lowly expressed in both THCA and LAML (Fig. 1B and Additional file 2: Table S2). We use a paired t-test as shown in Annex (Additional file 1: Figure S1).

To analyze the expression of *IDO1* at the protein level, we also assessed the IHC results from the HPA dataset and compared them to the TCGA data on *IDO1* gene expression. The results are shown in (Fig. 1C**)**. The six tumors analyzed with the protein level expression of *IDO1* showed higher expression of *IDO1* than normal tissues, which is consistent with the TCGA.

### Multifaceted prognostic value of *IDO1*

**Fig. 2:**
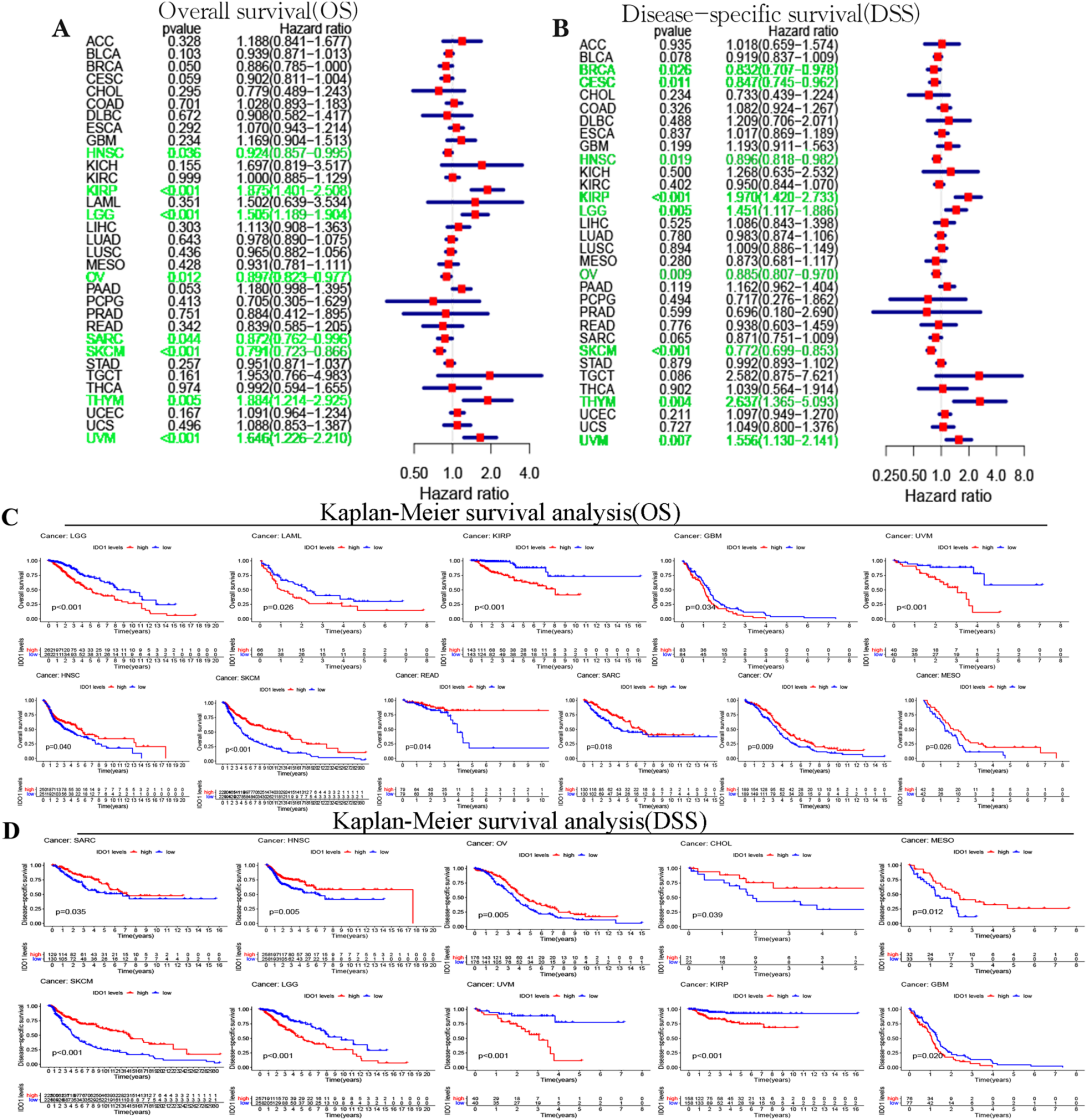
Relationship between the OS **A** and DSS **B** of patients and the expression level of IDO1. Red squares represent the hazard ratio. Kaplan–Meier survival curves of OS **C** and DSS **D** with comparison of high and low expression of IDO1 gene

**Fig. 3:**
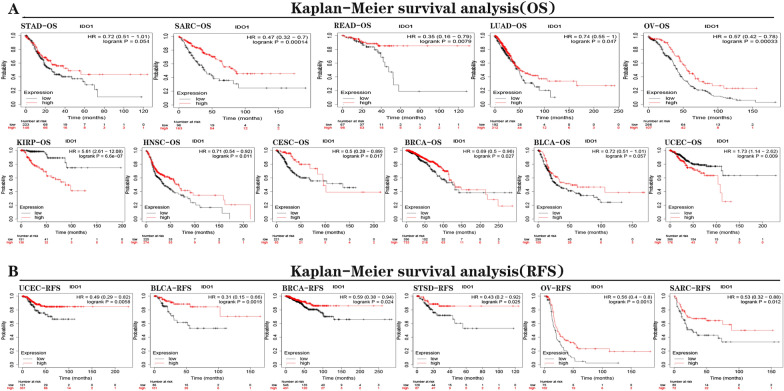
Kaplan–Meier survival curves in Kaplan–Meier Plotter. **A** OS of STAD, SARC, READ, LUAD, OV, KIRP, HNSC, CESC, BRCA, BLCA, and UCEC. **B** RFS of UCEC, BLCA, BRCA, STSD, OV, and SARC

We first used COX analysis (Fig. 2A–B and Additional file 1: Figure S2A–B)to investigate the association of *IDO1* with patients’ prognosis (OS, DSS, DFI, and PFI). Next, we used K-M analysis to investigate. For OS, *IDO1* exhibited an unfavorable prognostic factor for UVM, LGG, KIRP, GBM, and LAML. *IDO1* was a favorable prognostic factor in OV, READ, MESO, SARC, SKCM, and HNSC. For DSS, *IDO1* was an unfavorable prognostic factor in UVM, GBM, KIRP, and LGG; *IDO1* was a protective prognostic factor in OV, MESO, SARC, SKCM, HNSC, and CHCL ((Fig. 2C–D and Additional file 2: Table S4). About DFI, *IDO1* played an favorable prognostic factor in OV, SARC, , and BLCA (Additional file 1: Figure S2C). About PFI, *IDO1* played an adverse prognostic factor in UVM, LGG, GBM, KIRP, and THYM. *IDO1* played a protective prognostic factor in OV, MESO, SARC, SKCM, HNSC, BRCA, and CHOL (Additional file 1: Figure S2D); Subsequently, the prognostic value of *IDO1* in *IDO1*-related survival (OS and RFS) was determined using Kaplan Meier plotter analysis (Fig. 3). Interestingly, we were able to verify that *IDO1* had a protective prognostic role in BLCA (OS: *P* = 0.057; RFS: *P* = 0.0015), BRCA (OS: *P* = 0.027; RFS: *P* = 0.024), OV (OS: *P* = 0.00033; RFS: *P* = 0.0013), SARC (OS: *P* = 0.00014; RFS: *P* = 0.012), UCEC (RFS: *P* = 0.0058), CESC (OS: *P* = 0.017), HNSC (OS: *P* = 0.011), STAD (OS: *P* = 0.0054), LUAD (OS: *P* = 0.047), STSD (RFS: *P* = 0.025), and READ (OS: *P* = 0.0079). In contrast, *IDO1* expression had a detrimental effect on KIRP (OS: *P* = 6.6e−07) and UCEC (OS: *P* = 0.009).

### Clinical characteristics of *IDO1*

**Fig. 4:**
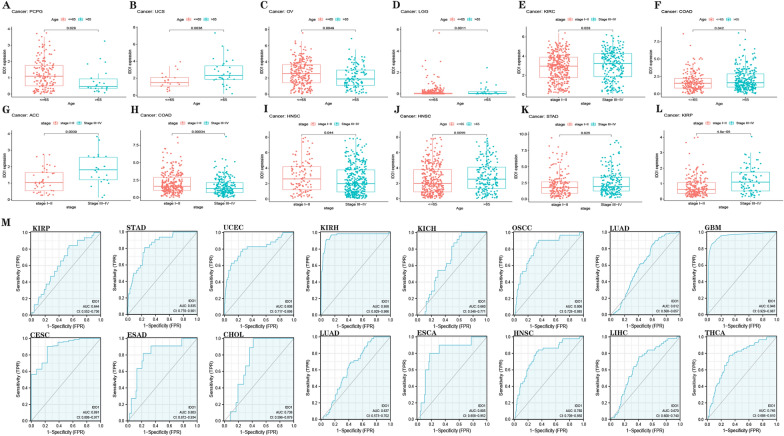
The relationship between IDO1 expression and age in PCPG **A**, UCS **B**, OV **C**, LGG **D**, and HNSC **E**. IDO1 expression related to the stage in COAD **F**, ACC **G**, COAD **H**, HNSC **I**, KIRC **J**, STAD **K**, and KIRP **L**. **M** the ROC curve of the IDO1 gene in cancers

According to Fig. 4A–L and Additional file 2: Table S3, a high level of *IDO1* expression was observed in patients under 65, especially in PCPG and OV. However, in UCS, LGG, COAD, and HNSC, *IDO1* expression was higher in patients older than 65 years. In addition, stage III-IV patients who had KIRC, STAD, ACC, and KIRP were highly expressed for IDO1, while stage I–II patients showed low expression for COAD and HNSC. Then, we plotted the ROC curve of the *IDO1* gene and associated cancers. The findings demonstrated that IDO1 had a moderate diagnostic accuracy (AUC between 0.6 and 0.9) for KIRP, STAD, UCEC, KICH, OSCC, LUAD, CESC, ESAD, CHOL, ESCA, HNSC, LIHC and THCA in predicting tumor or non-tumor prognosis, but a higher diagnostic accuracy (AUC > 0.9) for KIRH and GBM (Fig. 4M). This implies that IDO1 has a high degree of tumor prediction capacity.

### *IDO1* was correlated with TMB and MSI

**Fig. 5:**
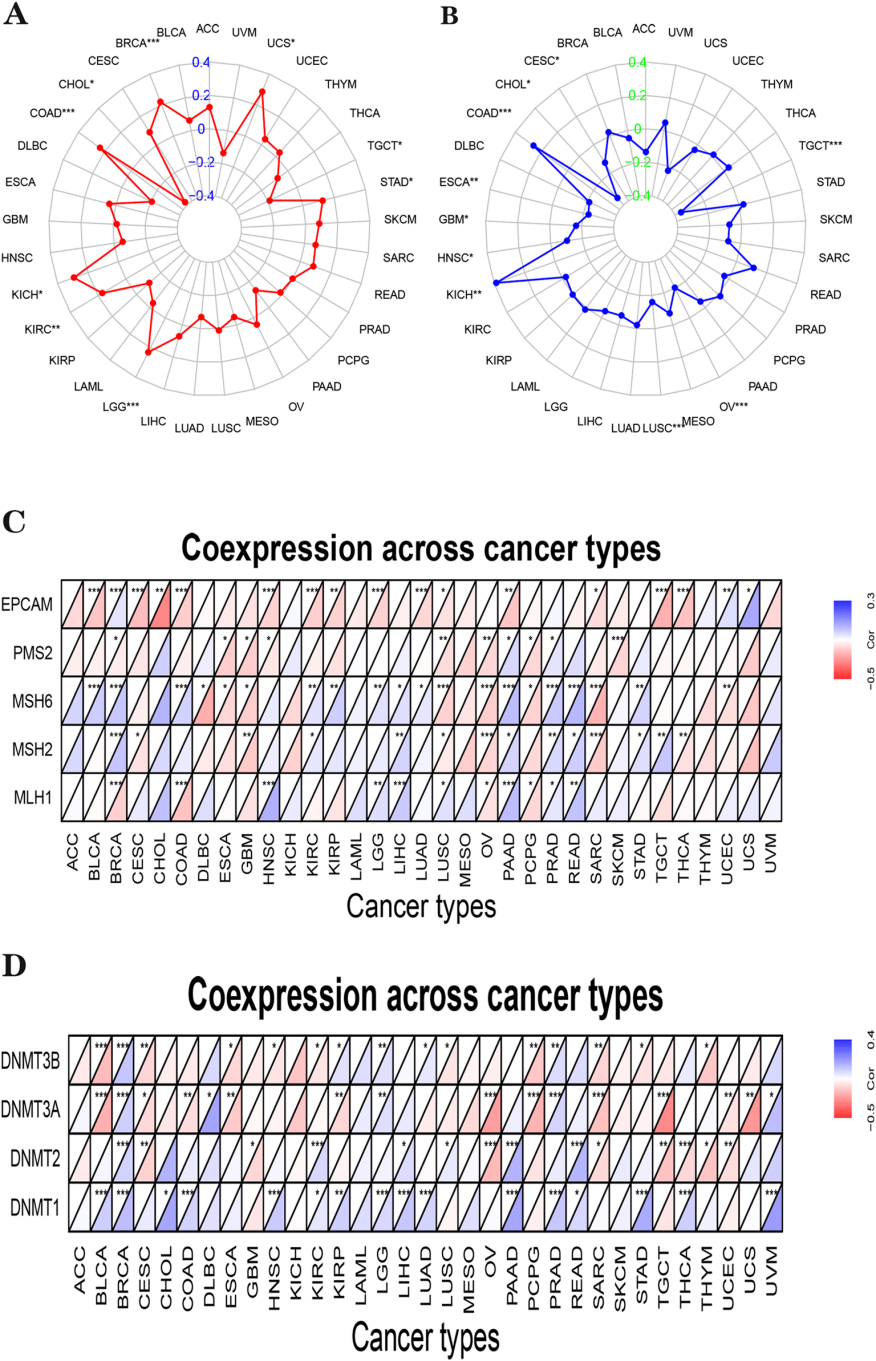
The radar chart of the relationships between the IDO1 and TMB **A** and MSI **B** in pan-cancer. Heatmap indicating the association between IDO1 expression and MMR **C** and DNMTs genes **D**

The TMB is a new biomarker correlated with immune checkpoint inhibitor sensitivity, containing PD-1/PD-L1 suppression [34–36]. Therefore, it is interesting to investigate the relation of *IDO1* expression with TMB. According to our findings, *IDO1* expression in nine tumors correlated with TMB, with positive correlations including UCS, STAD, KIRC, LGG, KICH, BRCA and COAD, and negative correlations in TGCT and CHOL (Fig. 5A). MSI is a hypermutation pattern generated by errors in the mismatch repair mechanism that has been employed as a marker for PD-1 Blockade [37–39]. The MSI status could alter the TME of cancer patients in several ways, thus influencing the prognosis. Therefore, we investigated the relationship between *IDO1* expression and MSI. The results of the study were shown by radar plots that among 10 different tumor types, only *IDO1* expression in KICH and COAD was positively correlated with MSI, while others including TGCT, OV, LUSC, HNSC, GBM, ESCA, CHOL, and CESC were negatively correlated (Fig. 5B). Among the 33 different tumors, IDO1 expression correlated with at least one MMR gene expression in 27 of them. Three tumors, BRCA, LUSC, and PAAD, showed a high correlation with the expression of MMR genes (Fig. 5C). Also, we investigated the connection between IDO1 and DNMT expression, which shows a correlation between 28 tumors and DNMT expression (Fig. 5 D).

### Correlation between pan-cancerous lymphocyte invasion and *IDO1* gene expression

**Fig. 6:**
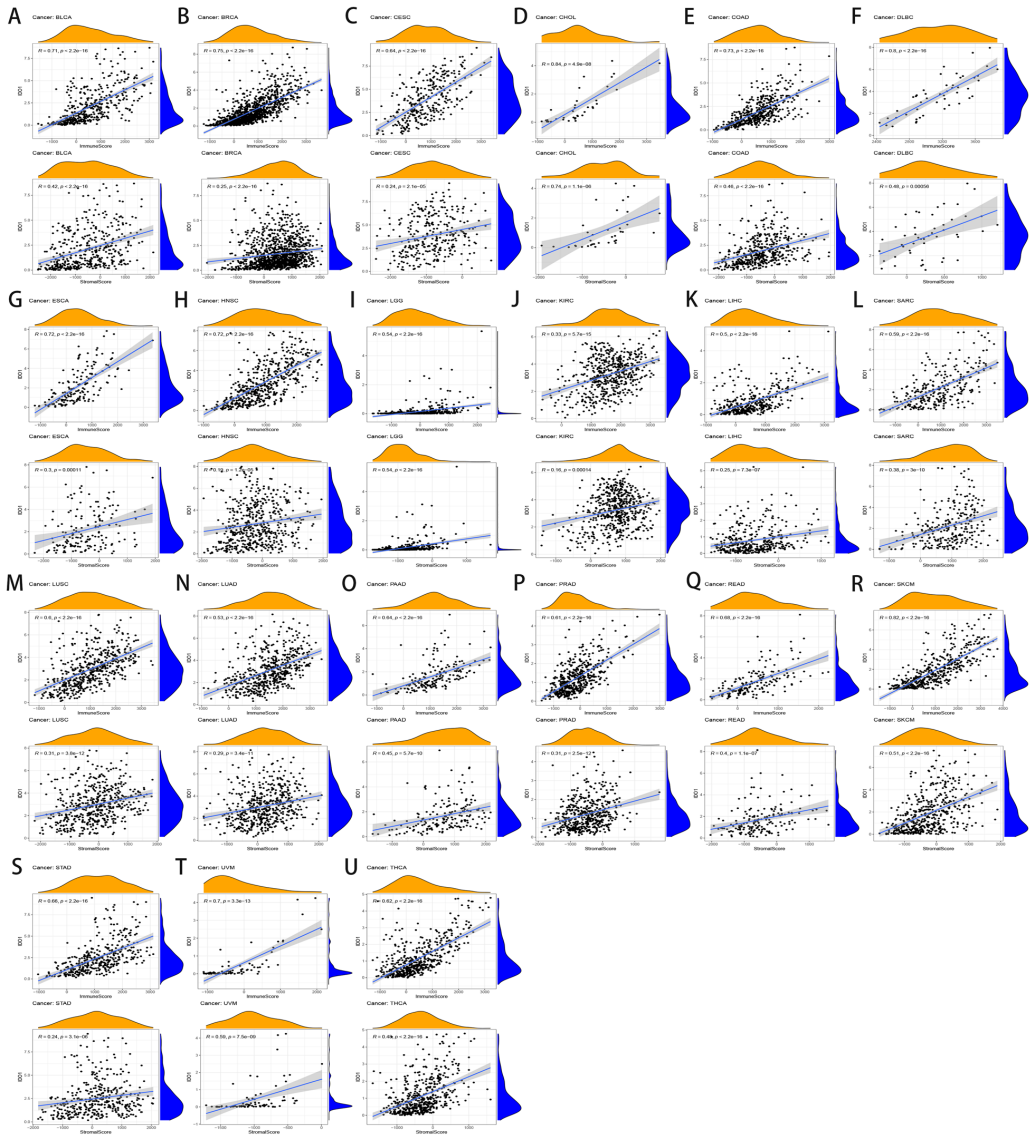
Correlation of IDO1 gene expression with stromal score and immune score in **A** BLCA; **B** BRCA; **C** CESC; **D** CHOL; **E** COAD; **F** DLBC; **G** ESCA; **H** HNSC; **I** LGG; **J** KIRC; **K** LIHC; **L** SARC; **M** LUSC; **N** LUAD; **O** PAAD; **P** PRAD; **Q** READ; **R** SKCM; **S** STAD; **T** UVM; **U** THCA

The TME plays a key role in promoting heterogeneity among tumor cells, thereby enhancing multidrug resistance and causing tumor progression and metastasis [40]. We sought to elucidate the connection between *IDO1* expression and immune infiltration since TIICs have been linked to the prognosis and treatment of many forms of cancer. We used the ESTIMATE method to explore the correlation between TME and *the IDO1* gene in different tumor tissues. We can see that stromal and immune cell level rises concurrently with an increase in *IDO1* expression in BLCA, BRCA, CESC, CHOL, COAD, DLBC, ESCA, HNSC, LGG, KIRC, LIHC, SARC, LUSC, LUAD, PAAD, PRAD, READ, SKCM, STAD, UVM, and THCA (Fig. 6A–U). Therefore, we believe that the expression of the *IDO1* gene may be related to the above tumors.

### Analysis of TIICs

**Fig. 7:**
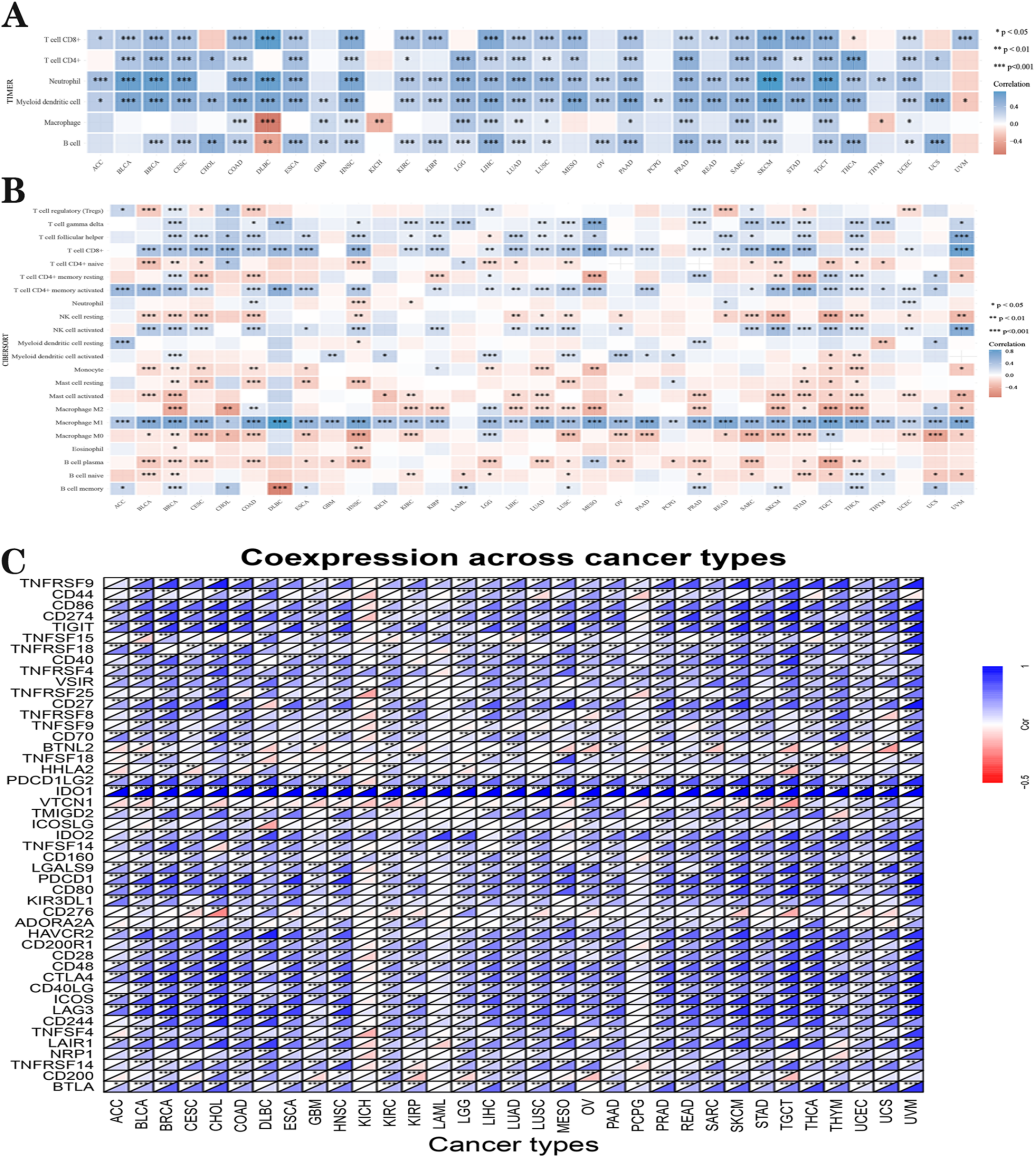
Research on TIICs and IDO1 expression was done using the **A** TIMER database and by the **B** CIBERSORT. **C** immune genes co-expression analysis

In cancer patients, biomarkers for checkpoint immunotherapy are key to improving prognosis [41]. Therefore, this study aims to explore the impact of *IDO1* on immunotherapy and explore the correlation between the expression of *IDO1* and the expression of immune checkpoints in 32 tumors. CD8^+^T cells are significantly positively correlated with the expression of *IDO1* in all tumors except CHOL, GBM, KICH, LGG, OV, PCPG, THYM, THCA, and UCS. CD4^+^ T cells were significantly positively correlated with the expression of *IDO1* in all tumors except ACC, DLBC, GBM, KIRP, KICH, OV, READ, PCPG, THYM, and UVM. Neutrophil cells were positively correlated with the expression of *IDO1* in all tumors except CHOL, GBM, KICH, PCPG, UCS, and UVM. Myeloid dendritic cells were positively correlated with the expression of *IDO1* in all but two tumors, KICH and THYM, where UVM was negatively correlated and all others were positively correlated. Macrophage was significantly positively correlated in COAD, GBM, HNSC, LGG, LUAD, LIHC, LUSC, PRAD, PAAD, SARC, TGCT, SKCM, and UCEC, but negatively correlated in DLBC, KICH, and THYM. And for B cells, except ACC, BLCA, KICH, MESO, PCPG, STAD, THYM, and UVM, *IDO1* is negatively correlated in DLBC, and positively correlated in all others. (Fig. 7A). We used R software CIBERSORT to analyze the connection between *IDO1* expression and the numbers of 22 TIICs. There is a significant correlation between the level of some TIICs and the expression of IDO1 in BRCA (*n* = 20), THCA (*n* = 17), LUSC (n = 15), COAD (*n *= 15), STAD (*n* = 15), TGCT (*n* = 14), HNSC (*n* = 14), CESC (*n* = 13), LGG (*n* = 13), BLCA (*n* = 12), UVM (*n* = 12), SKCM (*n* = 12), SARC (*n* = 12), LUAD (n = 11), and PRAD (*n* = 11) (Fig. 7B). *IDO1* is positively correlated with *CD274*, *TNFRSF9*, *TNFRSF4*, *PDCD1LG2*, *IDO2*, and *CD48* in many tumor types (Fig. 7C). Additionally, to better understand the connection between IDO1 and immunity, we used three methods to measure immune cells in the TME in pan-cancer, including EPIC, QUANTISEQ, MCP-counter (Additional file 1: Fig. S3).

### Drug sensitivity correlation analysis of *IDO1*

**Fig. 8:**
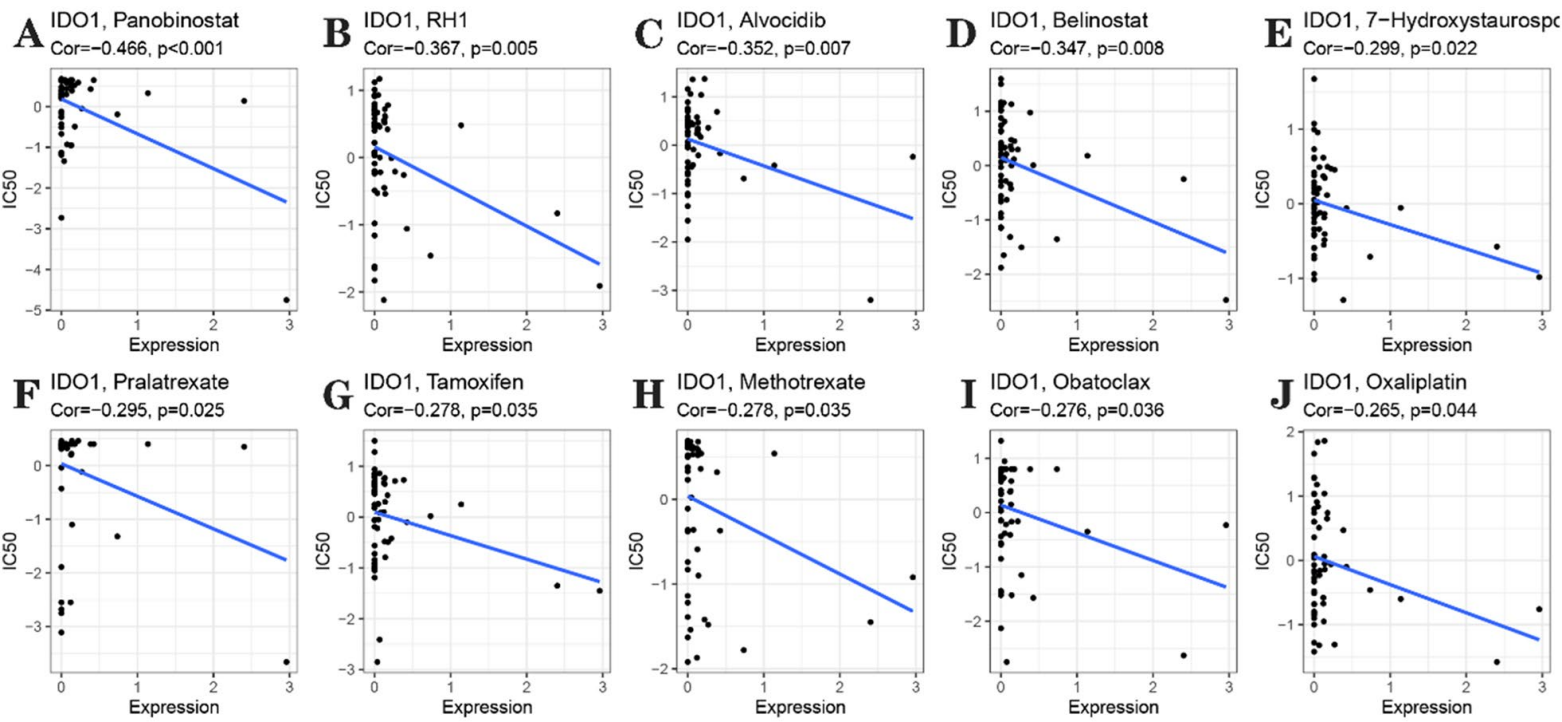
IDO1 expression was related to the sensitivity of Panobinostat **A**, RH1 **B**, Alvocidib **C**, Belinostat **D**, 7 − Hydroxystaurosporine **E**, Pralatrexate **F**, Tamoxifen **G**, Methotrexate **H**, Obatoclax **I**, and Oxaliplatin **J**

Further analysis of the potential correlation between drug sensitivity and IDO1 expression was conducted using the CellMiner TM database. The results showed that for the Panobinostat, RH1, Alvocidib, Belinostat, 7 − Hydroxystaurosporine, Pralatrexate, Tamoxifen, Methotrexate, Obatoclax, and Oxaliplatin they are all correlated negatively with the expression of *IDO1* **(**Fig. 8A–J**)**. According to the results, chemotherapy drugs including Panobinostat and Tamoxifen, which are frequently used in clinics, may be resistant to some chemotherapeutic agents because of *IDO1*.

### Functional analysis with GSEA

**Fig. 9:**
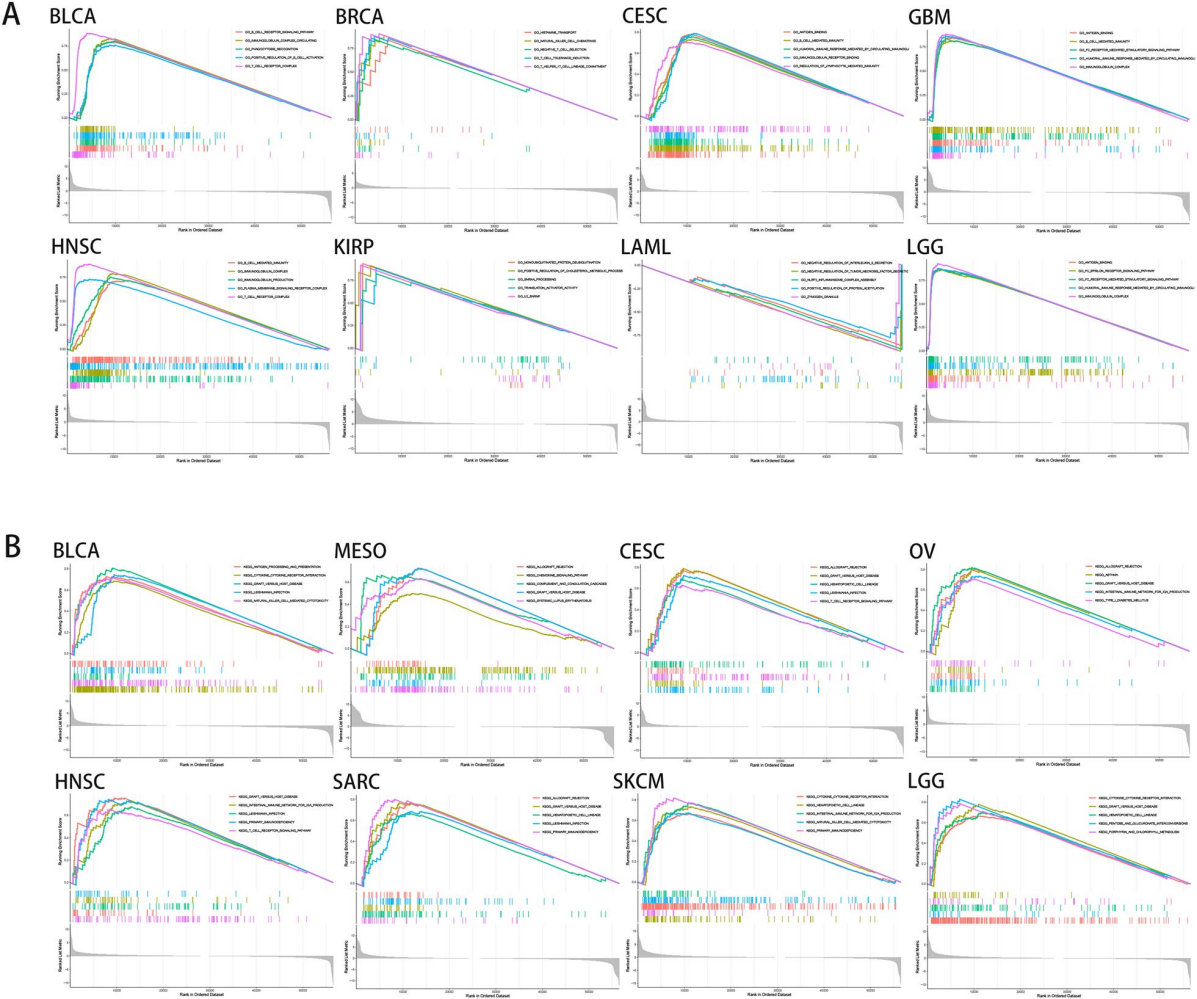
IDO1 pathway analysis in several cancer types. **A** Gene IDO1 functional annotation in GO **B** KEGG pathway analysis in various malignancies. Upward curve peaks indicate positive regulation while downward curve peaks indicate negative regulation

To look into the biological relevance of IDO1 expression in various tumor tissues, we analyzed the role of *IDO1* in various tumors by GO functional annotation and KEGG pathway. In GO, IDO1 has multiple bioactivities in BLCA, CESC, BRCA, GBM, KIRP, HNSC, LAML and LGG with diverse modulating effects on patient prognosis; for instance: T cell tolerance induction, regulation of lymphocyte-mediated immunity, immunoglobulin complex circulating, T cell receptor complex, negative regulation of tumor necrosis factor secretion, FC receptor-mediated stimulatory signaling pathway, B cell receptor signaling pathway (Fig. 9A). Among them, most pathways are strongly related to immunity or cancer. KEGG pathway analysis revealed that *IDO1* influenced signaling pathways in BLCA, MESO, CECS, OV, HNSC, SARC, SKCM, and LGG, such as cytokine receptor interaction, natural killer cell-mediated cytotoxicity, T cell receptor signaling pathway, chemokine signaling pathway (Fig. 9B). NK cell-mediated cytotoxicity, chemokine signaling pathway, T cell receptor signaling pathway, and other signaling pathways are a few of the signaling pathways connected to immunity or cancer. These routes and functions, as previously mentioned, mostly relate to tumors.

### *IDO1* expression level in immune subtypes

**Fig. 10:**
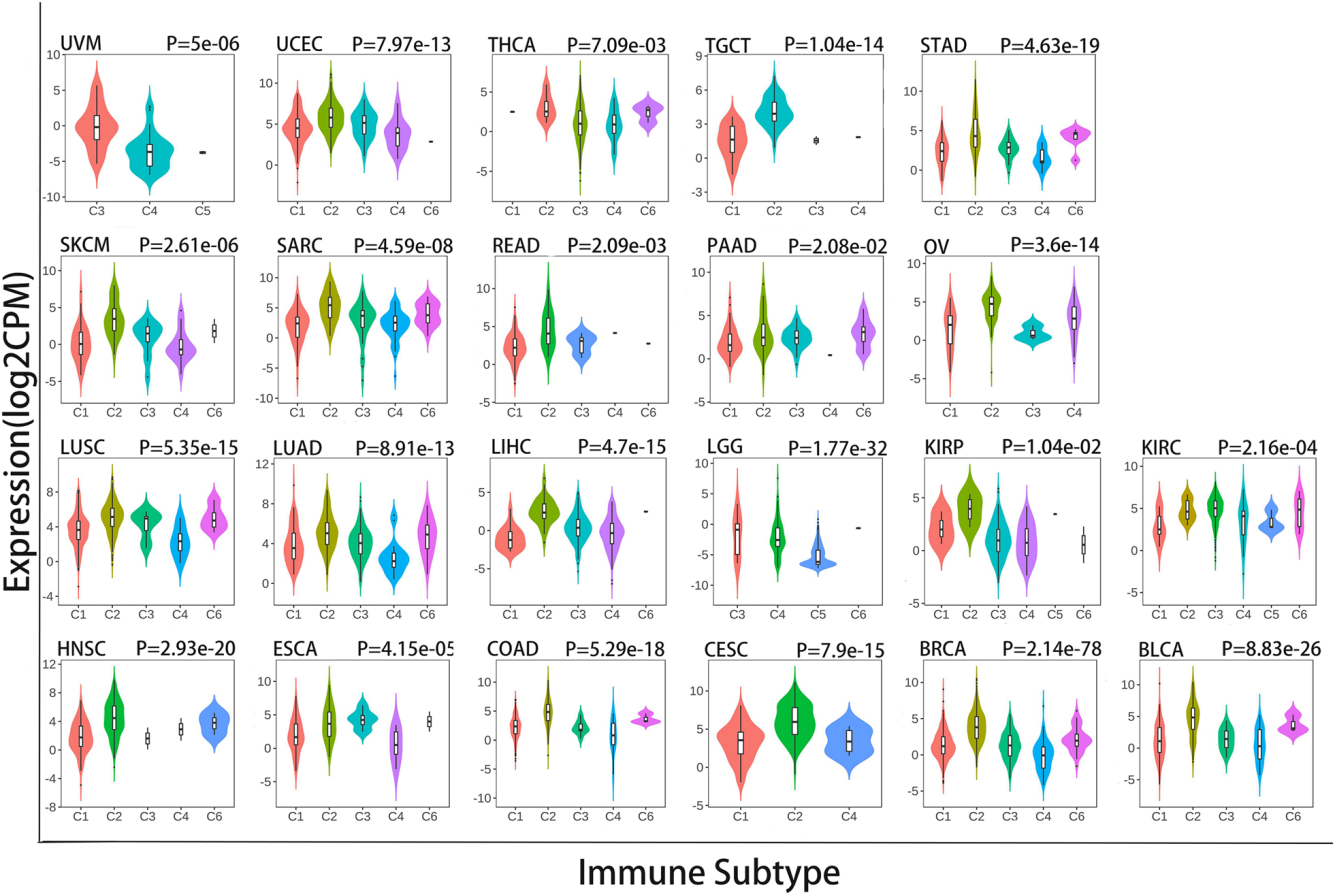
The TISIDB was used to determine the IDO1 expression levels in the immune subtypes in UVM, THCA, UCES, TGCT, SKCM, STAD, SARC, PAAD, READ, OV, LUAD, LUSC, LIHC, KIRP, LGG, KIRC, ESCA, HNSC, COAD, BRCA, CESC and BLCA

Based on our earlier findings, we discovered that OS in a range of malignancies is influenced by IDO1 expression levels, whether high or low. Consequently, we collected information on IDO1 expression from the TISDB website for the study of the link between molecular subtypes of the immune system and various tumors. The data demonstrated a substantial connection between IDO1 expression and immunological subtype of UVM, UCES, THCA, TGCT (uveal melanoma), STAD, SARC, SKCM, READ, OV, PAAD, LUSC, LIHC, LUAD, LGG, KIRC, KIRP, HNSC, COAD, ESCA, CESC, BRCA and BLCA (Fig. 10). Although there was no statistically significant difference in IDO1 expression between the immunological subtypes of UCS, MESO, KICH, GBM, ACC, and CHOL (data not shown).

### Expression and prognosis of IDO1

**Fig. 11:**
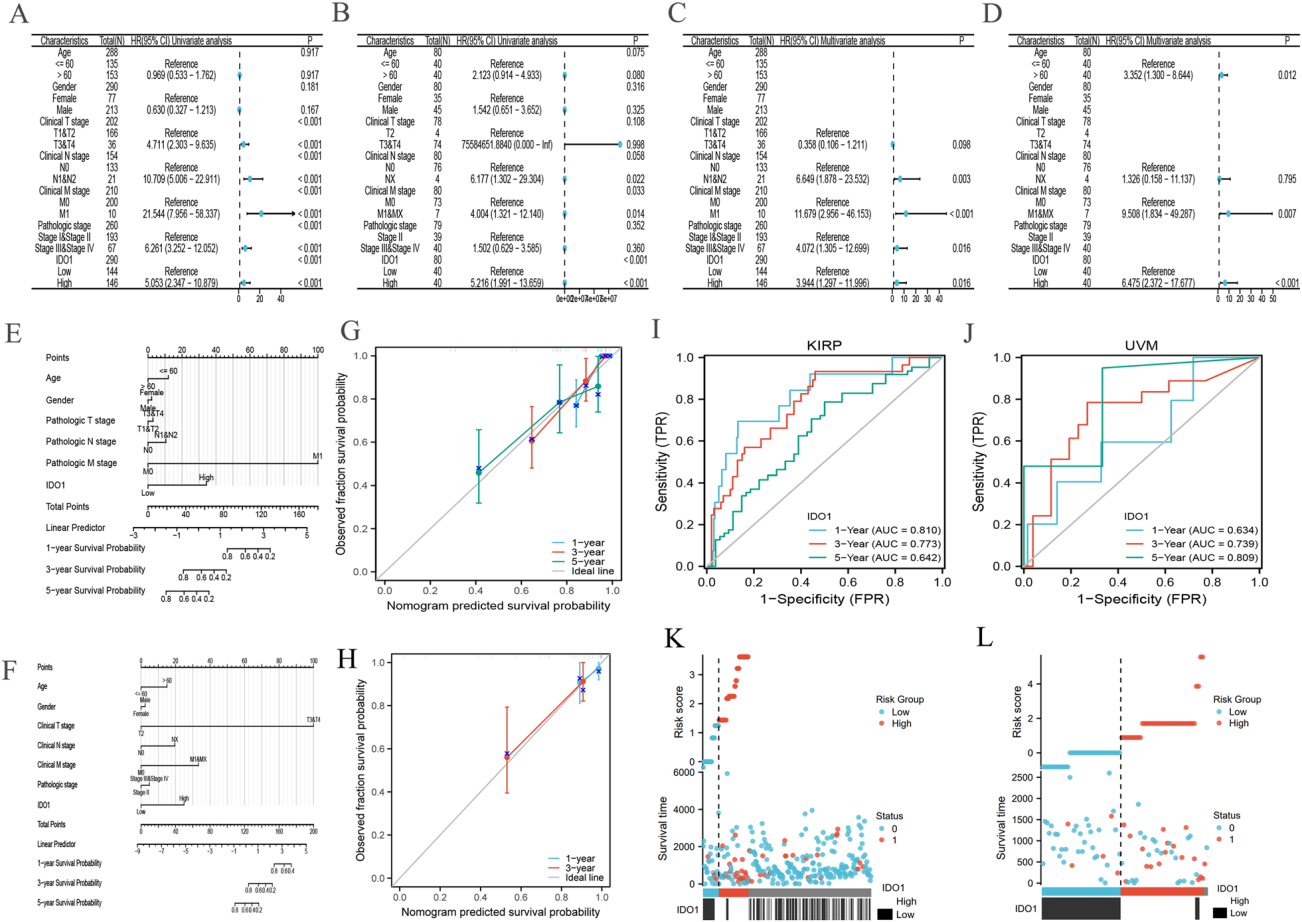
Univariate Cox regression analysis in KIRP **A**, UVM **B** and multivariate Cox analysis of KIRP **C**, UVM **D** including IDO1. Nomogram for KIRP **E**, UVM **F** for OS. Time-dependent ROC curves for OS prediction of KIRP **G** and UVM **H** patients. Plots of calibration data for KIRP **I** and UVM **J** OS predictions over one, three, and 5 years. The survival time distribution, risk score, and IDO1 expression of KIRP (**K**) and UVM (**L**) patients

We found that the factors we included were significantly associated with the OS of KIRP patients (Fig. 11A) and UVM patients (Fig. 11B). The risk factors were also included in multivariate Cox regression (Fig. 11C–D). According to the results, IDO1 is an independent prognostic factor for patients with KIRP and UVM.In the nomogram mode, the clinical characteristics of KIRP (Fig. 11E) and UVM (Fig. 11F) were incorporated. There was a good match between the predicted probability of calibration plots of KIRP (Fig. 11G) and UVM (Fig. 11H) and the results observed. Our research has developed calibration plots and time-dependent ROC curves predicting the odds of OS rates after 1 year, 3 years, and 5 years. The AUCs in terms of 1 year, 3 year, and 5 year were 0.810, 0.773, and 0.642 for KIRP patients, respectively (Fig. 11I). And for UVM patients, the statistics are 0.634, 0.739, 0.809, respectively (Fig. 11J). We also analyzed the correlation between risk score, survival time, and *IDO1* expression profiles of KIRP (Fig. 11K) and UVM (Fig. 11L) patients.

### Single-cell sequencing analysis of *IDO1* and PCR expression

**Fig. 12:**
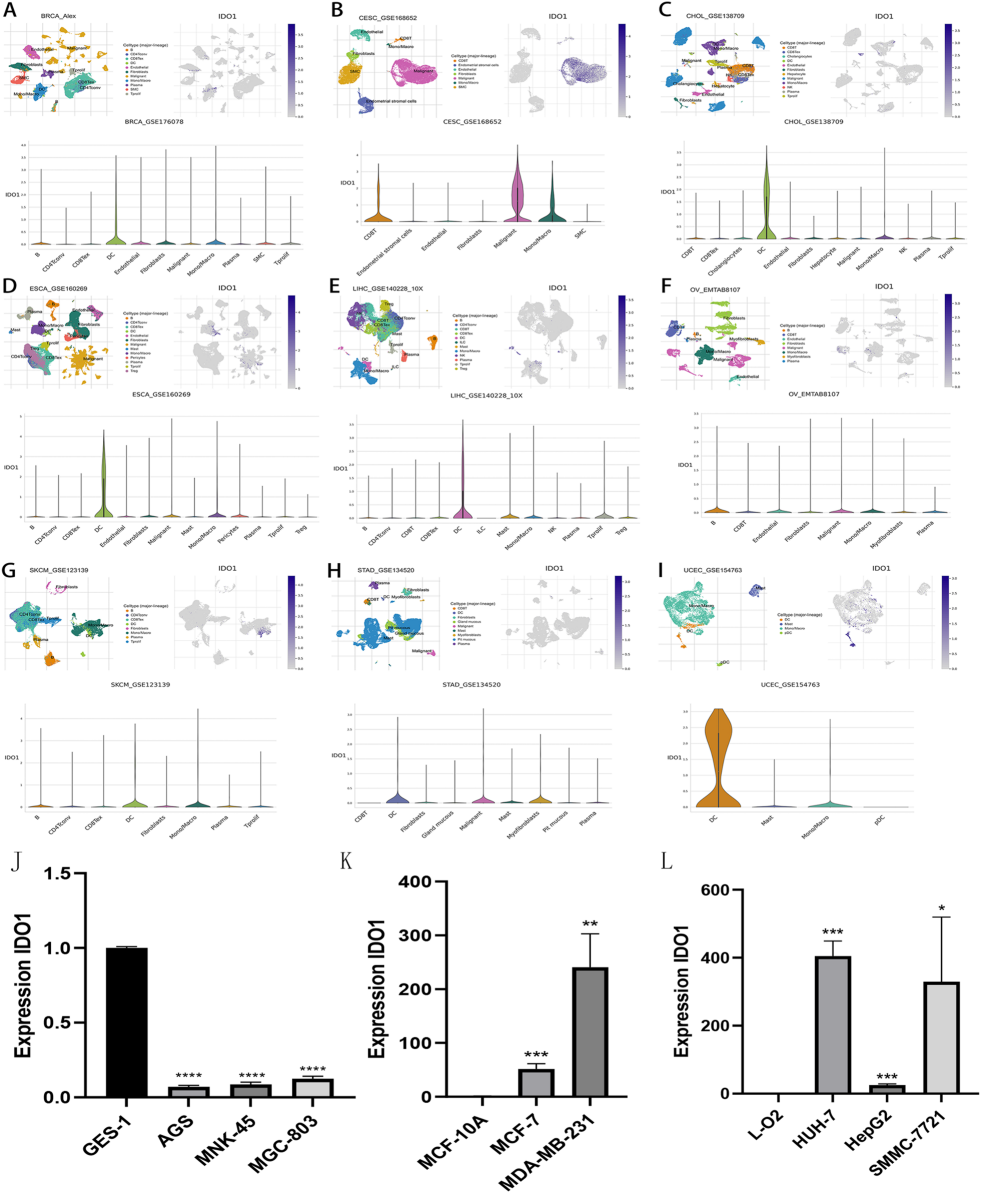
Pan-cancer single-cell sequencing analysis of *IDO1* co-expression in tumor cells and stromal cells and expression levels in different cancer cell lines. The levels of *IDO1* expression in tumor and stromal cells in BRCA **A**, CESC **B**, CHOL **C**, ESCA **D**, LIHC **E**, OV **F**, SKCM **G**, STAD **H** and UCEC **I**. **J** *IDO1* expression in gastric cell lines; **K** *IDO1* expression in breast cell lines; **L** *IDO1* expression in liver cell lines

Following that, the TISCH database was used for *IDO1*-related single-cell analysis. We looked at *IDO1* expression in tumor and stromal cells in several cancer types, including BRCA, CESC, CHOL, ESCA, LIHC, OV, SKCM, STAD, and UCEC (Fig. 12A–I). It’s interesting to note that the data showed that *IDO1* is extensively co-expressed on cancer cells and stromal cells in various malignancies, particularly on dendritic cells (DC), Malignant cells, Macrophages, and Endothelial cells. The PCR results showed that in contrast to normal cells, *IDO1* was poorly expressed in gastric cancer cells MKN-45, AGS, and MGC-803 by PCR (Fig. 12J). *IDO1* was found to be significantly highly expressed in breast cancer cells MCF-7 and MDA-MB-231 and hepatocellular carcinoma cells HepG2, HUH-7, and SMMC-7721 in comparison to normal cells (Fig. 12K–L). The results of PCR experiments are in line with the bioinformatics analysis.

## Discussion

Tumors as the number one disease threatening human health, and the goals of tumor immunology and immunotherapy are to enhance, expand, and forecast the therapeutic effectiveness of immune checkpoint inhibitor-based therapies [12]. Antitumor activity of targeted blockade of *IDO1* is remarkable, and *IDO1* is expected to be an emerging target for immune checkpoints [42]. In this study, we conducted bioinformatics analysis and laboratory experiments to understand the role and characteristics of *IDO1* in the immunology field of tumors.

First, we examined the *IDO1* expression in various tumors using the TCGA database as well as the TCGA in combination with the GTEX database. We found that *IDO1* expression was upregulated in ER-HER2 and HER2 + breast, colon, and endometrial cancers [43], thyroid cancer [44], gastric cancer [45], and gynecological tumors [46]. One factor contributing to the aggressiveness of GBM is the expression of the potent *IDO1* within tumors [47].

In terms of OS, *IDO1* expression was unfavorable in the five cancers (GBM, KIRP, LAML, LGG, and UVM) where *IDO1* expression was low, and gene expression was positively correlated with survival. Cancer cells overexpress *IDO1* which suppresses T cell effector function and promotes Tregs. In severe cases of cancer, overexpression of *IDO1* leads to poor survival [48]. However, it was a favorable factor in the six cancers (HNSC, MESO, OV, READ, SARC, and SKCM) where *IDO1* expression was high, and gene expression was negatively correlated with survival. Related studies have shown that for HNSC patients, *IDO1* was significantly higher before than after treatment and that patients with combined reduced levels of *PD-L1* and *IDO1* expression after treatment showed better PFS and OS [49].

Among women with HR + breast cancer (BC), high *IDO1* expression was associated with poorer long-term survival. The *IDO1* protein is present in most HR + BC and is an independent negative prognostic factor [50]. Trp is converted into downstream catabolites known as Kyn by *IDO1* [8]. Trp and Kyn depletion and increase exert immune functions through myeloid suppressor cells, and Treg [51]. Interferon-gamma is closely associated with *IDO1* expression in tumor treatment and prognosis. *IDO1* was primarily expressed in endothelial cells and positively correlated with IFN and T-cell [52]. *IDO1* overexpression and IFN-γ treatment increased Kyn accumulation and reduced tryptophan to induce autophagy more powerfully in cervical cancer cells [51]. *IDO1* and *PD-L1* expression in lung adenocarcinoma cell lines in the presence of IFN-γ and transforming growth factor-β were previously reported to be significantly linked with shorter DSS and OS [53]. Similarly, we have learned that in ESCC, prognosis and pathologic response were negatively affected by increased IDO1 expression in tumors [54]. CD40 mAb combined with epacadostat, an IDO1 inhibitor, reduced tumor growth in B16-F10 melanoma, accompanied by an increase in tumor-infiltrating T cells [52]. Therefore, focusing on the differential expression of *IDO1* in different tumors and its prognosis in comparison with combination drug therapy, *IDO1* could be a good immune checkpoint to start developing *IDO1* inhibitor-related therapeutics to improve anti-tumor efficacy and patient survival.

Currently, immunotherapy has shown remarkable results in the fight against malignant tumors [55]. Our research revealed that TME is crucial in promoting interstitial cancer cells, which promotes the growth and spread of cancer cells, and raises treatment resistance. *IDO1* is highly expressed and promotes metastasis, drug resistance, cell proliferation, and TAM resistance through STAT3 and interleukin-6 in TAM-resistant breast cancer, [56]. In many tumors, the transcription factor AHR of growth-promoting genes is activated by KYN in parallel with *IDO1* and KYN, enhancing CD28 expression and survival signaling [57]. Targeting KYN can, therefore, be used as a pathway for tumor immunotherapy. Successful immunotherapy for the treatment of malignancies is severely hampered by the immunosuppressive milieu created by Treg [58]. Treg-mediated increased glucose intake causes cellular senescence and decreases the response to T cells through cross-talk. Human Treg cells’ inhibition of glucose uptake and glycolysis as a result of TLR8 receptor signaling results in increasing anti-tumor immunity after adoptive transfer T cell therapy for melanoma [58].

Moreover, TIICs play an important anti-tumor role in TME. In the current investigation, we discovered that the levels of different TIICs, such as B cells, phagocytes, CD8T cells, CD4T cells, bone marrow dendritic cells, and NK cells, were closely linked with the levels of *IDO1* expression in different cancers. The binding of CD80/CD86 on dendritic cells to CD28 on LLPC activates *IDO1* [57] and promotes the production of the catabolic product Kyn, indirectly affecting the *IDO1*/TDO2-Kyn-AhR [21] signaling pathway. For the regulatory axis *IDO1*/miR-18a/NKG2D/NKG2DL in the tumor microenvironment, cytotoxicity of NK cells regulated with high expression of *IDO1* was significantly reduced, regulating NK cell function [59]. According to the previous study, *IDO1* expression has been shown to be positive for tumor B-cell infiltration [50]. By comparing related studies, we can confirm that IDO1 expression has a positive effect on TIICs.

Next, co-expression analysis reveals that *IDO1* was highly correlated with multiple immune-related gene expressions in pan-cancer. Meanwhile, based on GO and KEGG enrichment analysis, we also found that *IDO1* was involved in numerous immune mechanisms and related pathways, including T cell tolerance induction, regulation of lymphocyte-mediated immunity, immunoglobulin complex circulating, T cell receptor complex, negative regulation of tumor necrosis factor secretion, FC receptor-mediated stimulatory, B cell receptor signaling pathway signaling pathway cytokine receptor interaction, T cell receptor signaling pathway, natural killer cell-mediated cytotoxicity, chemokine signaling pathway. In summary, the differential expression of *IDO1* was closely related to tumor immunity and may serve as a novel target for immunotherapy and prognostic markers.

The drawback of our current study is that further information from other open datasets is required to validate and corroborate our findings. Also, there are not enough experiments to support this, a larger sample size is needed to validate the role and mechanism of IDO1 in pan-cancer.

## Conclusion

The findings of this study reveal that *IDO1* has abnormal expression in a number of malignancies and is related to the prognosis for UVM, LGG, KIRP, GBM, LAML, OV, READ, MESO, SARC, SKCM, and HNSC. Furthermore, across a variety of cancer types, the aberrant *IDO1* expression was connected to the TMB, MSI, MMR, medication sensitivity, and TIME. These results offer a more solid theoretical foundation for the possibility that IDO1 could be a useful prognostic biomarker and a possible indicator of immunotherapy sensitivity in a range of cancers. The clinical treatment of IDO1 is now better supported by a theoretical basis and guidelines provided by our study.

### Supplementary Information

**Additional file 1: Figure S1.** *IDO1* expression in contrast between paired normal and non-tumor specimens. **Figure S2.** The forest maps of *IDO1* expression level with survival in different cancers. **Figure S3.** Research on TIICs and *IDO1* expression was done using the **A** EPIC,** B** , **C** MCP-counter methods

**Additional file 2: Table S1.** Expression levels of IDO1 comparing tumor and normal tissues from TCGA database. **Table S2.** Expression levels of IDO1 comparing tumor and normal tissues from TCGA and GTEx database. **Table S3.** Clinical annotation and Pathological features of the individual patient in TCGA database. **Table S4.** Expression levels of IDO1 comparing tumor and normal tissues in K-M survival curves(OS).

## Data Availability

The raw data of this study are freely available from the website TCGA Research Network (https://portal.gdc.cancer.gov/), GTEx(http://commonfund.nih.gov/GTEx/), TIMER database(https://cistrome.shinyapps.io/timer/), Kaplan–Meier plotter portal(https://kmplot.com/analysis/), cBioPortal databasehttp://cbioportal.org), and HPA (https://wwwproteinatlas.org/). All the analyzed data are included in the manuscript.

## Abbreviations

IDO1: Indoleamine2,3-dioxygenase1
PD-1: Programmed death-1
PD-L1: Programmed cell death 1 ligand 1
TCGA: The Cancer Genome Atlas
GTEx: Genotypic tissue expression project
TMB: Tumor mutation burden
MSI: Microsatellite instability
MMR: Mismatch repair
DNMTs: DNA methyltransferases
GO: Gene ontology
KEGG: Kyoto Encyclopedia of Genes and Genomes
TIGIT: T cell immune receptor with Ig and ITIM domains
CTLA4: Cytotoxic T Iymphocyte associate protein-4
OS: Overall survival
DFS: Disease-free survival
ACC: Adrenocortical carcinoma
BLCA: Bladder urothelial carcinoma
BRCA: Breast invasive carcinoma
CESC: Cervical squamous cell carcinoma and Endocervical adenocarcinoma
CHOL: Cholangiocarcinoma
COAD: Colon adenocarcinoma
DLBC: Lymphoid neoplasm diffuse large B-cell lymphoma
ESCA: Esophageal carcinoma
GBM: Glioblastoma multiforme
HNSC: Head and Neck squamous cell carcinoma
KICH: Kidney chromophobe
KIRC: Kidney renal clear cell carcinoma
LAML: Acute myeloid leukemia
LGG: Brain lower grade glioma
LIHC: Liver hepatocellular carcinoma
LUAD: Lung adenocarcinoma
LUSC: Lung squamous cell carcinoma
MESO: Mesothelioma
OV: Ovarian serous cystadenocarcinoma
PAAD: Pancreatic adenocarcinoma
PCPG: Pheochromocytoma and paraganglioma
PRAD: Prostate adenocarcinoma
READ: Rectum adenocarcinoma
SARC: Sarcoma
SKCM: Skin cutaneous melanoma
STAD: Stomach adenocarcinoma
TGCT: Testicular germ cell tumors
THCA: Thyroid carcinoma
THYM: Thymoma
UCEC: Uterine corpus endometrial carcinoma
UCS: Uterine carcinosarcoma
UVM: Uveal melanoma
