# Supplementary material for: Comprehensive analysis of the prognostic value and immunological role of IDO1 gene in pan-cancer
Source: Eur J Med Res. 2024 Mar 27;29:206. doi: 10.1186/s40001-024-01766-y (PMC10967207; doi:10.1186/s40001-024-01766-y)
Supplement: Supplementary file 1 — Additional file 1: Figure S1. IDO1 expression in contrast between paired normal and non-tumor specimens. Figure S2. The forest maps of IDO1 expression level with survival in different cancers. Figure S3. Research on TIICs and IDO1 expression was done using the A EPIC, B , C MCP-counter methods [file 40001_2024_1766_MOESM1_ESM.docx]

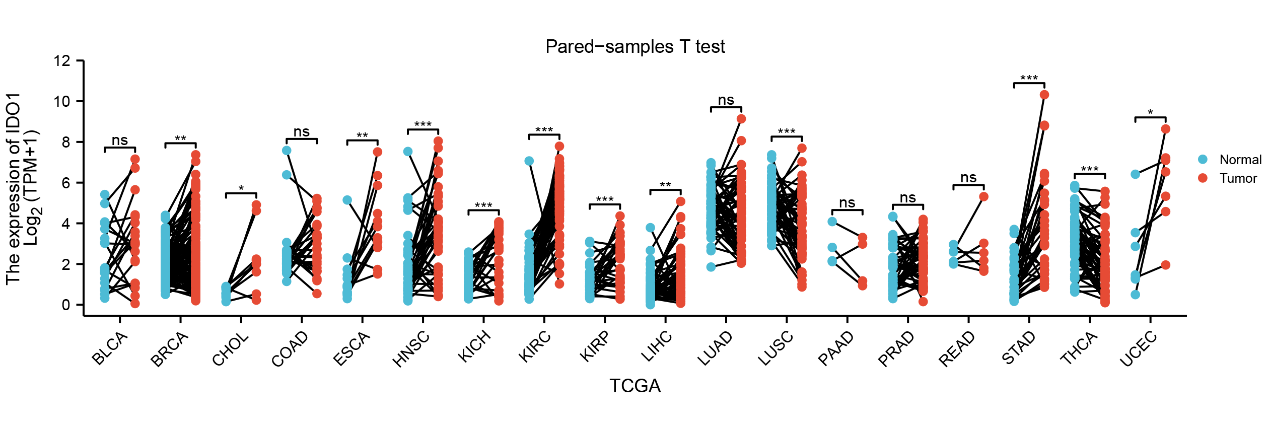


**Figure s-1** *IDO1* expression in contrast between paired normal and non-tumor specimens.


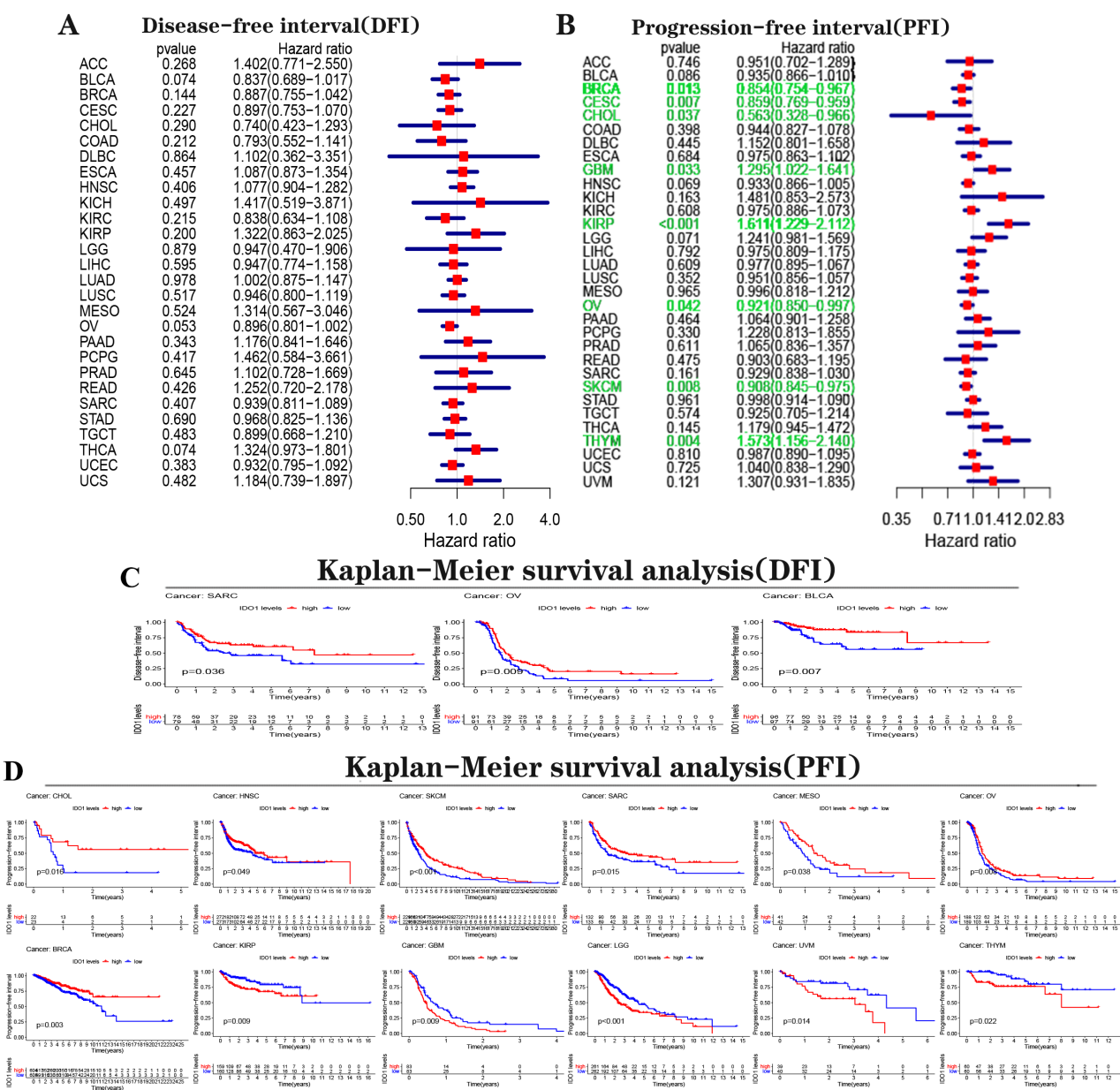


**Figure s-2.** The forest maps of *IDO1* expression level with survival in different cancers. Association between *IDO1* expression level and patients’ DFI (A), PFI (B). Red squares represent the hazard ratio. K-M survival curves with high and low expression of *IDO1* gene in pan-cancer in K-M Plotter of DFI (C) and PFI (D).
